# Participation and engagement in online cognitive testing

**DOI:** 10.1038/s41598-024-65617-w

**Published:** 2024-06-26

**Authors:** Daniel Radosław Sokołowski, Jasmine Pani, Tor Ivar Hansen, Asta Kristine Håberg

**Affiliations:** 1 Department of Neuromedicine and Movement Science, Faculty of Medicine and Health Sciences, Norwegian University of Science and Technology, Fred Kavli Building, 3rd floor, south, Olav Kyrres gate 9, 7030 Trondheim, Norway; 2 MiDT National Norwegian Research Center, St. Olav’s University Hospital, Trondheim, Norway

**Keywords:** Neuropsychology, Selection bias, Internet, Adolescents, Lifespan, Neuroscience, Psychology, Diseases, Medical research, Risk factors, Signs and symptoms

## Abstract

Web-based testing of cognitive abilities allows for large-scale assessments without geographical constraints. Yet, the extent to which it can reach populations beyond the typical demographic groups recruited for cognitive studies is unclear. This study focused on comparing the characteristics of individuals from a general population study (HUNT4) who chose to participate in a cognitive study (HUNT4-Hjernetrim) with those who did not. Additionally, we investigated participants' engagement and user experience. We obtained data on socio-demographics, health conditions (both physical and mental), self-reported cognitive or learning difficulties, and lifestyle factors of Hjernetrim participants and non-participants from the HUNT4 database. Hjernetrim involved 13 cognitive tests, administered through the online platform Memoro. We used logistic regressions to assess participation biases and linear regressions to assess participants’ engagement and user experience. Of 65,851 HUNT4 participants invited via regular mail to Hjernetrim, 5634 (9.4%, aged 13–97, 54% women) participated. The best represented in the sample were 50–79-year-olds, women, tertiary educated, living alone, from urban areas, not occupationally active, and reporting memory complaints. Individuals who were aged 80+, had motor or vision impairments, and teenagers with learning disabilities, were underrepresented. Participants were more likely to have mental health problems, have or survived cancer and less likely to have cardiovascular disease. Participants logged on mainly during weekdays, the preferred time of day varied by age. On average, participants used 42 min and completed 78% of the tasks. Using PCs provided the most complete data. In terms of user experiences, 65% were positive while 14% were negative or reported technical difficulties. Overall, the study demonstrated that web-based methodology allowed for a relatively well-represented sample that included groups typically difficult to reach. The presence of somatic and mental diseases had a variable influence on participation. Participants finished most tests and reported positive experiences overall.

## Introduction

Web-based testing of cognitive functions allows for geographically unconstrained and large-scale assessment of cognition without the need for participants to install specific software, use a particular device, or travel to a testing location. The method has several advantages over in-person or phone-based assessments^1^. For instance, the methodology is significantly more cost-effective than traditional pen-and-paper testing^2^ and allows participants to complete tests at their convenience, in a location of their choice. Moreover, participants often prefer this administration form over traditional testing, stating they feel more in control and are free of being scrutinized by a test administrator^3^, leading to a more comfortable test experience. The increasing familiarity with desktop computers and mobile devices across all age groups^4^ along with the expanding access to the internet around the world (internetworldstats.com/stats4.htm, datareportal.com/reports/digital-2022-global-overview-report) sets the stage for the successful integration of web-based cognitive testing. This holds particular promise for epidemiological studies.

Epidemiological studies have been experiencing declining participation rates in recent decades^5–7^. The implementation of a web-based methodology may increase participation by providing more flexibility, but possibly also introduce new types of bias^8,9^. The latter could stem from factors such as willingness and/or ability to engage in self-administered testing and varied computer familiarity, as well as specific health problems.

Since selection bias is a concern for the representativeness of results from epidemiological studies, understanding how web-based data collection affects participation is a relevant and timely undertaking. Yet, few studies address selection bias in the context of web-based data collection. This issue is not unique to web-based methodologies, as exemplified by a census analysis of research articles from the Cancer Epidemiology, Biomarkers & Prevention journal, which revealed that only 44% of published studies documented response rates, approximately 10% compared participants to non-participants, and among longitudinal studies, only 3 out of 17 reported dropout rates^10^. The studies that have investigated participation biases show numerous differences between participants and non-participants. A previous study performed on the Trøndelag Health Study (The HUNT Study) sample showed participants to have higher levels of education and better health than non-participants^11^. Furthermore, the mode of assessment, e.g. web-based testing, can introduce bias, as indicated by studies that have compared web-based surveys to paper-based and mixed-mode surveys and identified variations in response rates and disparities in performance^9,12^. A specific concern related to web-based cognitive testing pertains to the administration of studies in non-laboratory settings. Participants use their own diverse devices at a time of their choosing, can discontinue at any time, and are not shielded from distractions.

In the current study, we used data from HUNT4, the fourth wave of the Trøndelag Health study (The HUNT study), a large general population study of the northern part of Trøndelag county and three districts of Trondheim, the largest city in the southern part of Trøndelag, Norway^5,13^, combined with information from the Hjernetrim substudy of HUNT4. In Hjernetrim, HUNT4 participants were invited by mail to take a self-administered, web-based cognitive test battery administered via Memoro^3,14^ assessing several aspects of cognitive abilities, including memory (pattern separation, working memory, spatial and verbal memory), perceptual speed, attention, and executive functions.

The current study has two main aims. Firstly, to characterize those who choose to participate in web-based cognitive testing. The objective is to compare the characteristics of individuals who participated in the HUNT4-Hjernetrim cognitive substudy with those who did not, with regard to sociodemographics, self-reported health, cognition, and lifestyle. We hypothesize that there are significant differences in the demographic, health, and lifestyle profiles between participants and non-participants.

Secondly, we aim to describe the participants’ engagement with the study. The objective is to assess: (1) their computer familiarity and preferred device and browser; (2) the time of day and week participants took the test; (3) the time spent completing the tasks; (4) the number of tasks completed and reasons for discontinuing; (5) participation at field station; and (6) participants’ self-reported user experience and frequency at which they accessed their performance results. We hypothesize that there are age-related variation in all these aspects of participant engagement.

Together, the results can be utilized by researchers to enhance their planning, design, and recruitment strategies, thereby improving participation rates and quality of data in future studies that employ web-based technologies.

## Materials and methods

### Study population and procedure

#### The HUNT4 study

The HUNT4 study is the fourth wave of the Trøndelag Health Study, one of the world's largest ongoing longitudinal population-based health studies^13^. The HUNT study is considered representative of Norway geographically and economically with age, morbidity, and mortality distribution similar to the national averages. HUNT4 data collection was approved by the Norwegian Data Inspectorate and Regional Committee for Research Ethics (REK-2016/801). Informed consent was obtained from all subjects and/or their legal guardian(s) and the study was performed in accordance with relevant guidelines and regulations, including the Declaration of Helsinki.

The research sample consisted of three geographical and age-based cohorts: the HUNT4-N (NT4) cohort consisted of all residents aged 20 years and older in the northern part of Trøndelag County, the Young-HUNT4 (YH4) cohort consisted of residents of the same area between ages 13 and 19, and finally the ST1 cohort consisted of adults aged 70 years and older living in three districts of Trondheim, the largest city in the region.

The three cohorts filled out mostly overlapping cohort-specific questionnaires, where some questions were adapted to the age group (e.g., adults were asked about cognitive issues while the youth were asked about learning disabilities). From the HUNT4 study the following variables were acquired: age (calculated from the date of birth to the day of invitation to NT4, YH4, or ST1), biological sex, education level, living alone or with a partner, work status, place of residence (rural vs. urban), general health issues and specific diagnoses, including scores on the Hospital Anxiety and Depression Scale (HADS), self-reported cognitive or learning problems, lifestyle satisfaction, and current smoking and alcohol use (Table 1). The scoring of the HADS was categorized into two groups: (1) scores below were considered within the normal range; (2) scores of 11 or greater indicated a potential clinical range of symptoms of anxiety and depression (fhi.no/globalassets/dokumenterfiler/rapporter/2016/maleegenskaper-ved-den-norske-versjonen-pdf.pdf). A detailed description of all variables included in the study can be found on the HUNT Databank website (hunt-db.medisin.ntnu.no/hunt-db).

**Table 1:** Description of the variables of interest and in which cohorts they were obtained.

Variable	Cohorts	Scale	Description and notes
Age	YH4, NT4, ST1	Continuous	Age at the invitation to HUNT4, calculated by subtracting the birthdate registered in the Norwegian National Registry from the date of sending an invitation to the HUNT4 study
Biological sex	YH4, NT4, ST1	Nominal, Man/Woman	Obtained from Norwegian National Registry
Education	YH4, NT4, ST1	Ordinal, 3 values	Stratified into 3 categories: Primary: Primary and lower secondary school, 7–10 years depending on birth cohort; Secondary: Academic, vocational school or apprenticeship, 1–4 years; Tertiary: Bachelor or higher education
Living alone or with a partner	NT4, ST1	Nominal, 2 values	“Do you live with someone?”: 1. No, I live alone; 2. Yes, spouse or partner
Occupationally active	NT4	Nominal, no/yes	“Are you occupationally active?”
Place of living: urban or rural	YH4, NT4, ST1	Nominal, 2 values	Obtained from Norwegian National Registry
Chronic illness or injury	NT4, ST1	Nominal, no/yes	“Do you suffer from longstanding (at least 1 year) illness or injury of a physical or psychological nature that impairs your functioning in your daily life?”
Conditions and diseases: myocardial infarction, diabetes, stroke or brain haemorrhage, cancer	NT4, ST1	Nominal, no/yes	“Have you had, or do you have any of the following diseases?” Myocardial Infarction, Diabetes, Stroke or Brain Haemorrhage, Cancer
Mental health problems	NT4, ST1	Nominal, no/yes	“Have you had, or do you have mental health problems you sought help for?”
HADS ≥ 11	NT4, ST1	Nominal, 2 values	The Hospital Anxiety and Depression Scale total score; or extrapolated score if 11, 12, or 13 out of 14 questions are answered. Stratified into: 1. HADS < 11; 2. HADS ≥ 11
Impairments: motor impairment, vision impairment, hearing impairment	YH4, NT4, ST1	Ordinal, 4 values	YH4: “Do you have impairments of your functioning in any of the following areas?” Motor Impairment, Vision Impairment, Hearing Impairment NT4 and ST1: “If yes [longstanding illness that impairs your functioning], would you describe your impairment as slight, moderate or severe?”: 1. Not impaired; 2. Slightly impaired; 3. Moderately impaired; 4. Severely impaired
Health	YH4, NT4, ST1	Ordinal, 4 values	”How is your health at the moment?”: 1. Poor; 2. Not so good; 3. Good; 4. Very good
Learning disabilities: any, math, reading/writing, other	YH4	Nominal, no/yes	“Do you have any learning disabilities? If yes, which disabilities?”
Memory problems	NT4, ST1	Ordinal, 3 values	“Do you have problems with your memory?”: 1. No; 2. Yes, some; 3. Yes, a lot
Lifestyle satisfaction	NT4, ST1	Ordinal, 4 values	“How satisfied are you with your lifestyle (diet, exercise, smoking and drinking habits)?” 1. Very satisfied; 2. Satisfied; 3. Less satisfied; 4. Not satisfied
Smoking	YH4, NT4, ST1	Nominal, 5 values	Smoking status: 1. Never smoked; 2. Ex daily smoker; 3: Current daily smoker; 4. Current occasional smoker; 5. Ex occasional smoker
Alcohol drinking	NT4, ST1	Nominal, no/yes	“Do you drink alcohol?”

In addition, the HUNT Databank includes data on age and sex from the Norwegian National Registry, on all those residing in the catchment area where the HUNT4 study was performed, i.e., also those who did not participate in HUNT4, which we obtained.

#### The HUNT4 Hjernetrim study

The HUNT4 Hjernetrim Study (hereafter called Hjernetrim) was accepted as a HUNT4 substudy by the HUNT4 planning committee in 2015. The data collection was approved by the Norwegian data inspectorate study as part of HUNT4, the Regional Committee for Research Ethics (REK-155024 HUNT4 Hjernetrim study), and the HUNT administration. The participants gave their informed consent online before the testing started. The study was performed in accordance with relevant guidelines and regulations, including the Declaration of Helsinki.

As required by the HUNT Research Centre, only participants who had taken part in the main HUNT4 study could be invited to Hjernetrim. An invitation letter for the Hjernetrim study was included in the feedback letter and sent by regular mail to participants from the three cohorts (NT4, YH4, ST1), totalling 65,851 invited individuals (54% women), of whom 5634 participated (54% women). The letter contained the results of the clinical assessment and physical activity accelerometer results collected in HUNT4 and personal health recommendations. The letter also included a half-fold 10.5 cm by 14.5 cm leaflet providing a brief description of the Hjernetrim study, its website address, and the participant-specific login credentials required to log on to the website. Participants were given instructions to manually enter this web address and their login credentials into their web browsers to access the web-based cognitive test platform, Memoro, for participation. Invitation letters were sent out continuously by regular mail as the results from the HUNT4 data collection were processed. The possibility of participating in Hjernetrim was additionally advertised in local newspapers, by flyers at shopping malls, and at the HUNT4 field stations.

Participants were told to set aside about 40 min to complete the tests, be in a quiet place, and preferably use a personal computer with the Google Chrome browser, although other platforms and browsers were also supported.

The Hjernetrim participation was conducted using our proprietary, validated, self-administered, web-based cognitive test platform Memoro^3,14^ to perform a selection of tests covering several domains. A total of 13 tests were administered in a fixed order: Simple Reaction Time, Pattern Separation, Visual Memory (Immediate Recall), Verbal Memory (Learning and Immediate Recall), Symbol-Digit Coding (Main Test), Symbol-Digit Coding (Recall of a Symbol-Digit Key), Digit Span Forward, Visual Memory (Delayed), Visual Memory (Recognition), Digit Span Backwards, Verbal Memory (Delayed Recall), Verbal Memory (Recognition), Complex Reaction Time.

Before cognitive testing started, the participants consented online and filled in a short questionnaire including birth year, gender, level of education, handedness, and computer familiarity assessed on a 5-point Likert scale (“How comfortable are you with computers?”). We used gender, age in years, and level of education (three levels, see above) to calculate normalized scores for participant-specific feedback on performance on the battery (see below).

The instructions for tasks were both written and verbally presented. The main tasks were preceded by short training sessions which participants needed to perform correctly in order to proceed to the main tasks. If training results indicated that task instructions were not understood, participants were automatically transferred to the next test.

For participants not familiar with using computers or not owning one, Hjernetrim field stations were arranged and advertised together with Hjernetrim information. Those who chose to participate in the field stations could use the computer equipment and technical assistance of the researchers available on-site and could log in using either their original credentials or, to keep the subject anonymous, use temporary credentials which were later connected back to their HUNT ID.

Participants who completed at least one test could access a feedback page that was available after completing or aborting the last task. Participants needed to log in anew to access the feedback. The mode of feedback was developed in collaboration with the HUNT administration and their user group before data collection started. The feedback page displayed details regarding the cognitive skills evaluated in Memoro, along with practical examples of how these abilities are employed in daily life. Participants could see how well they performed on the different tasks in comparison to others of the same gender, age, and education group rated from the participant’s best to the worst test performance and for each test whether the performance was above, at, or below the participant’s age, gender and education group average.

### Engagement, user experience, and feedback

We collected metadata on Hjernetrim participation for each log-in. The metadata included device type, operating system, web browser type and version, number of logins, the date and time of starting and finishing each of the tasks, and the status of each task (started, finished, aborted, failed).

For participants logging more than once and using multiple devices, we defined the participant’s main device and web browser engine as the ones that were used to complete the highest number of tasks. For participants who completed an equal number of tasks on more than one device and/or web browser, we used the one that was used first.

The time and day of participation were registered at the first login to Memoro. To analyze them, we defined one day as the period between 4 and 4 am of the next day to separate the “night owls” from the “early birds”. If a participant completed the battery in more than one session, only the first session was considered.

We calculated battery completion time as the sum of the time taken to complete the training and main tasks from start to finish, measured in minutes, excluding the breaks that participants took between the tasks. Tasks in which participants spent less than 5 times the interquartile range (IQR) or more than 10 times the IQR were considered outliers and excluded from the analysis.

We defined the number of finished tasks as the sum of main tasks that were not aborted by participants or as a result of failed training, and that provided valid scores. Training tasks were not included in the battery completion statistics.

Participants who did not start all tasks were categorized as having discontinued the battery. Successful completion of all 13 tasks was not necessary to avoid being classified as having discontinued; participants only needed to initiate each task.

After the testing session concluded, participants were asked to indicate whether they had encountered any disruptions or interruptions during one or more tasks. They could do this by marking the affected tasks off on a provided list. They were also asked to share their overall experience with the testing in the form of an open question “Please, tell us about your experience”. The free text responses were subsequently stratified into the following categories: general positive experience (e.g. “Fun”); general negative experience (e.g. “Stressful”); comments about battery difficulty or own performance (e.g. “Struggled with word task, was better with numbers”); any technical issues with the platform (e.g. “Aborted a task by mistake”, “Sound problems”, “Images did not appear”); and other comments (typically about a specific task, like its length, instructions, or about being interrupted, e.g. "Disturbed by dad ", “Difficult, especially with a screaming baby”).

Contact information for the researchers (e-mail address and phone number) was available in the invitation mail and on the Memoro website for participants who experienced technical issues, wanted to participate in a field station, or had other inquiries.

### Statistical analysis

Participants’ sociodemographic and health characteristics, as well as metadata from the web testing, are presented as frequencies and percentages or means and standard deviations, as appropriate.

#### Participation bias

To examine factors affecting the likelihood of participation in Hjernetrim, we compared those participating in HUNT4 but not in Hjernetrim (n = 60,217) and those participating in both studies (n = 5634) as the dependent variable in logistic regression. For each variable, the group with the largest number was used as the reference group when reporting the odds ratios (OR). First, we examined age groups in decades, sex, and education (stratified into primary, secondary, and tertiary). In all subsequent logistic regression models, age group, sex, and education were included as variables of no interest. We investigated other sociodemographic variables next, namely living alone or with a partner, work status, and place of residence (urban or rural). Subsequently, we investigated health-related variables: general health and impairment(s), and the presence of specific somatic and mental diagnoses/problems. Then we investigated the impact of cognitive and learning issues. Next, the impacts of satisfaction with own lifestyle, smoking, and alcohol use were assessed.

Finally, as age and sex were available for participants from all invited to HUNT4 (n = 119,558) including those who did not participate and thus were not invited to the Hjernetrim study, we used it to assess the effects of age and sex on the likelihood of participation in Hjernetrim among the general population. We used a logistic regression model with participation as a dependent variable and age group and biological sex as variables of interest.

#### Engagement in Hjernetrim

To assess engagement among those participating in HUNT4 Hjernetrim, we used linear regression models with the time of day and weekday of starting the battery, time spent in the battery, and number of finished tasks as the dependent variable in separate models with age or age group, sex, education, and, where appropriate, device used and computer familiarity, as independent variables.

To assess factors leading to discontinuation and leaving feedback, we used logistic regressions with discontinuation and leaving feedback as the dependent variable in separate models. In both models, the independent variables were age group, sex, education level, device used, and computer familiarity. For the model analyzing leaving feedback, we additionally included the number of tasks completed by the participants as an independent variable.

To assess the level of computer familiarity across Hjernetrim participants, we used ordered logistic regression with computer familiarity as a dependent variable and age group, sex, and education as independent variables.

For dummy variables, the most numerous subgroup from HUNT4 for each variable was used as the reference group when reporting odds ratios (OR).

## Results

### Sample characteristics

**Figure 1:**
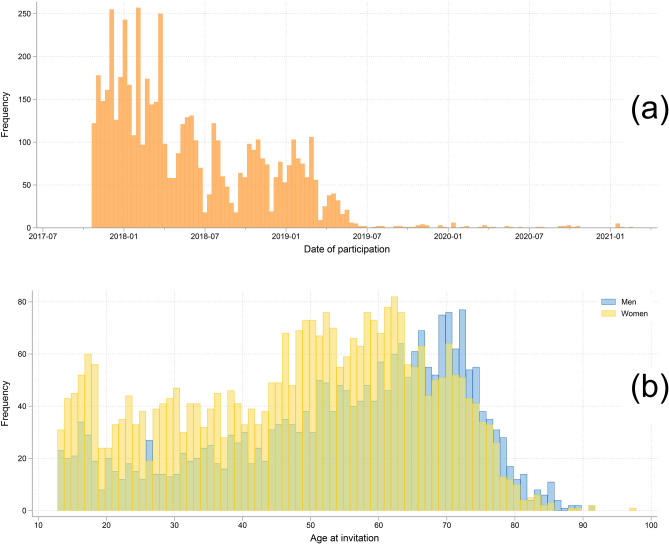
(**a**) Date of participation (year-month) in HUNT4 Hjernetrim (frequency = n). (**b**) Age and sex distribution of the Hjernetrim participants.

**Figure 2:**
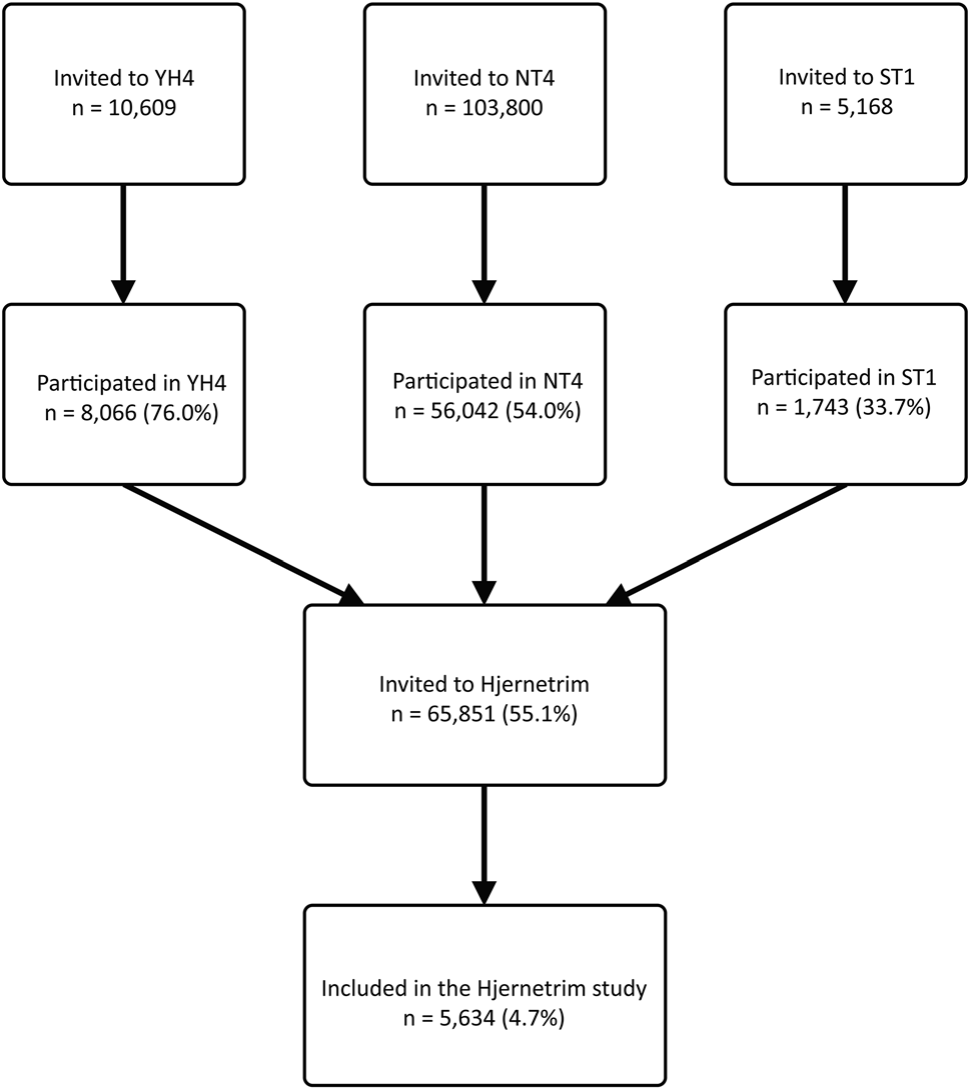
Flowchart showing the number of Hjernetrim participants with the number of people invited to the substudy and entire cohorts.

Hjernetrim data were collected during the HUNT4 study between November 2017 and February 2021, with most of the data (69%) collected in 2018 (Fig. 1a). Overall, 5,634 participants (3,254 women, 2,380 men, 13 to 97 years old, Fig. 1b) gave consent to participate in Hjernetrim, which translates into 8.6% of those invited and 4.7% of the general population (Fig. 2). An overview of the sociodemographic, health, cognitive, and lifestyle-related variables in the HUNT4-only participants and those who also participated in Hjernetrim are presented in Table 2.

**Table 2:** Sociodemographic and health characteristics for those participating in the HUNT4 study who did not participate in Hjernetrim and those participating in both the HUNT4 study and Hjernetrim.

Variable [*f* (% of n)]		HUNT4 n = 61,419		Hjernetrim n = 5634	
		Women	Men	Women	Men
Participants		33,138 (54.0%)	28,282 (46.0%)	3254 (57.8%)	2380 (42.2%)
Age group^a^	13–19	4118 (12.4%)	4107 (14.5%)	312 (9.6%)	156 (6.6%)
	20–29	3191 (9.6%)	2568 (9.1%)	352 (10.8%)	159 (6.7%)
	30–39	3506 (10.6%)	2695 (9.5%)	389 (12.0%)	215 (9.0%)
	40–49	4602 (13.9%)	3639 (12.9%)	508 (15.6%)	290 (12.2%)
	50–59	5155 (15.6%)	4529 (16.0%)	674 (20.7%)	437 (18.4%)
	60–69	5137 (15.5%)	4854 (17.2%)	631 (19.4%)	594 (25.0%)
	70–79	4655 (14.0%)	4112 (14.5%)	353 (10.8%)	465 (19.5%)
	80 +	2774 (8.4%)	1778 (6.3%)	35 (1.1%)	64 (2.7%)
Education	Primary	5834 (18.3%)	4315 (15.7%)	231 (7.2%)	154 (6.5%)
	Secondary	14,700 (46.0%)	15,231 (55.5%)	1217 (37.7%)	975 (41.3%)
	Tertiary	11,408 (35.7%)	7921 (28.8%)	1778 (55.1%)	1231 (52.2%)
Living with a partner		19,795 (76.8%)	18,146 (82.4%)	18,146 (82.4%)	1742 (83.9%)
Occupationally active		15,760 (59.4%)	14,172 (63.5%)	1963 (69.9%)	1311 (63.5%)
Place of living: urban		18,937 (66.3%)	15,194 (64.6%)	2164 (73.4%)	1660 (74.6%)
Chronic illness or injury: no		15,213 (54.3%)	13,704 (57.9%)	1774 (61.0%)	1396 (63.4%)
Myocardial infarction: no		26,411 (97.8%)	21,462 (93.5%)	2850 (99.1%)	2042 (94.6%)
Diabetes: no		30,020 (95.0%)	25,386 (93.4%)	3075 (96.1%)	2183 (93.9%)
Stroke or brain haemorrhage: no		26,006 (96.8%)	21,810 (95.8%)	2806 (97.7%)	2053 (95.6%)
Cancer: no		24,864 (91.8%)	21,072 (91.8%)	2644 (91.9%)	1950 (90.6%)
Mental health problems: no		21,353 (79.0%)	20,114 (88.1%)	2174 (75.6%)	1838 (85.8%)
HADS < 12^b^		15,901 (71.5%)	12,678 (75.8%)	1968 (73.6%)	1501 (77.7%)
Motor impairment	No impairment	7784 (57.2%)	6923 (57.9%)	793 (65.1%)	448 (54.8%)
	Slight	3494 (25.7%)	2975 (24.9%)	304 (25.0%)	243 (29.7%)
	Moderate	1596 (11.7%)	1421 (11.9%)	98 (8.1%)	93 (11.4%)
	Great	734 (5.4%)	631 (5.3%)	23 (1.9%)	34 (4.2%)
Vision impairment	No impairment	5830 (42.6%)	5822 (49.2%)	576 (47.4%)	366 (45.0%)
	Slight	5484 (40.0%)	4393 (37.1%)	476 (39.2%)	361 (44.4%)
	Moderate	1820 (13.3%)	1266 (10.7%)	125 (10.3%)	75 (9.2%)
	Great	567 (4.1%)	346 (2.9%)	37 (3.1%)	12 (1.5%)
Hearing impairment	No impairment	8689 (63.2%)	6328 (52.2%)	829 (67.9%)	399 (47.8%)
	Slight	3188 (23.2%)	3478 (28.7%)	274 (22.4%)	288 (34.5%)
	Moderate	1171 (8.5%)	1533 (12.6%)	78 (6.4%)	98 (11.7%)
	Great	707 (5.1%)	789 (6.5%)	40 (3.3%)	50 (6.0%)
Health	Poor	546 (1.72%)	434 (1.6%)	45 (1.4%)	28 (1.2%)
	Not very good	7644 (24.1%)	5083 (18.6%)	583 (18.2%)	386 (16.5%)
	Good	18,188 (57.4%)	16,682 (61.0%)	1868 (58.4%)	1394 (59.6%)
	Very good	5336 (16.8%)	5163 (18.9%)	702 (22.0%)	532 (22.7%)
Any learning disabilities: no		2770 (75.1%)	2664 (73.8%)	232 (82.3%)	113 (81.3%)
Math disabilities: no		3178 (86.2%)	3204 (88.8%)	259 (91.8%)	130 (93.5%)
Reading/writing disabilities: no		3276 (88.9%)	3063 (84.9%)	257 (91.1%)	123 (88.5%)
Other disabilities: no		3407 (92.4%)	3380 (93.7%)	266 (94.3%)	134 (96.4%)
Memory	No problems	9222 (46.8%)	7198 (47.1%)	1140 (49.0%)	877 (49.2%)
	Some problems	9917 (50.3%)	7683 (50.3%)	1139 (49.0%)	873 (49.0%)
	Large problems	565 (2.9%)	393 (2.6%)	47 (2.0%)	32 (1.8%)
Lifestyle satisfaction	Very satisfied	2535 (9.1%)	2448 (10.4%)	286 (9.8%)	278 (12.6%)
	Satisfied	19,482 (69.9%)	16,705 (71.1%)	2003 (68.4%)	1558 (70.9%)
	Not very satisfied	5051 (18.1%)	3885 (16.5%)	549 (18.7%)	322 (14.6%)
	Not satisfied	792 (2.8%)	473 (2.0%)	91 (3.1%)	40 (1.8%)
Smoking	Never smoked	13,229 (45.5%)	10,947 (44.0%)	1378 (46.1%)	981 (43.7%)
	Ex occasional smoker	2951 (10.2%)	2749 (11.0%)	401 (13.4%)	268 (11.9%)
	Ex daily smoker	9598 (33.0%)	8773 (35.3%)	980 (32.8%)	851 (37.9%)
	Current occasional smoker	501 (1.7%)	703 (2.8%)	54 (1.8%)	43 (1.9%)
	Current daily smoker	2767 (9.5%)	1714 (6.9%)	176 (5.9%)	102 (4.5%)
Current alcohol drinker		17,509 (78.5%)	14,707 (87.5%)	2287 (85.8%)	1753 (90.7%)
Computer familiarity [mean (SD)]^c^		–	–	4.4 (0.8)	4.4 (0.8)

Results are provided as numbers and percentages.

^a^Measured at the invitation to Memoro.

^b^Hospital Anxiety and Depression Scale Total Score.

^c^Computer familiarity was measured in Memoro participants only.

### Sociodemographic variables associated with HUNT4 participants partaking in Hjernetrim

#### Demographics

**Figure 3:**
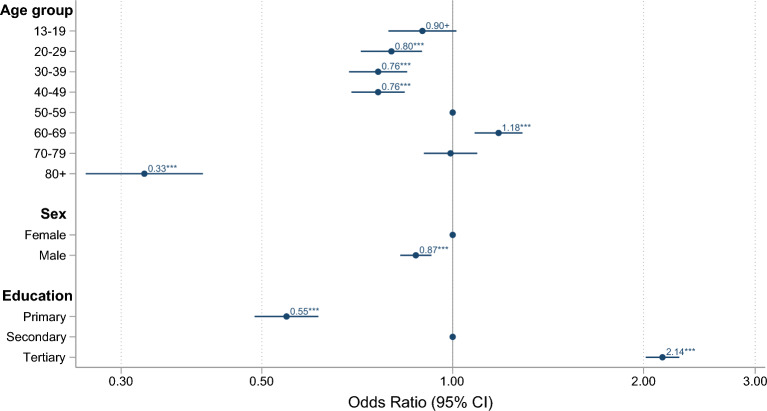
The odds ratio of age group, sex, and education on participation in Hjernetrim relative to participation in HUNT4. Fifty-year-olds, women, and those with secondary education were used as references as these were the most numerous subgroups. For details on variables and their frequency, see Tables 1 and 2.

Age, sex, and education affected the likelihood of participation in Hjernetrim (Fig. 3). Compared to 50-year-olds, the 60–69-year group was the most likely to participate in Hjernetrim (OR = 1.18, 95% CI [1.08, 1.29]), whereas participants ≥ 80-year were the least likely (OR = 0.33, 95% CI [0.26, 0.40]). Sex distribution differed across age, with more women (62.9%) participating in Hjernetrim below age 65, but more men (56.3%) among those aged 65 and older (Fig. 1b). Overall, men were less likely to participate (OR = 0.87, 95% CI [0.83, 0.93]). The probability of participating was positively associated with educational attainment, with those with only primary education least likely (OR = 0.55, 95% CI [0.49, 0.61]) and those with tertiary education most likely to participate (OR = 2.14, 95% CI [2.02, 2.28]).

Living alone increased the participation likelihood (OR = 1.13, 95% CI [1.04, 1.22]) compared to living with a spouse or partner. Not being occupationally active also increased OR of participation (OR = 1.10, 95% CI [1.01, 1.20]). Living in a rural area decreased the likelihood of participation (OR = 0.73, 95% CI [0.68, 0.78]).

#### Somatic and mental health variables associated with partaking in Hjernetrim

**Figure 4:**
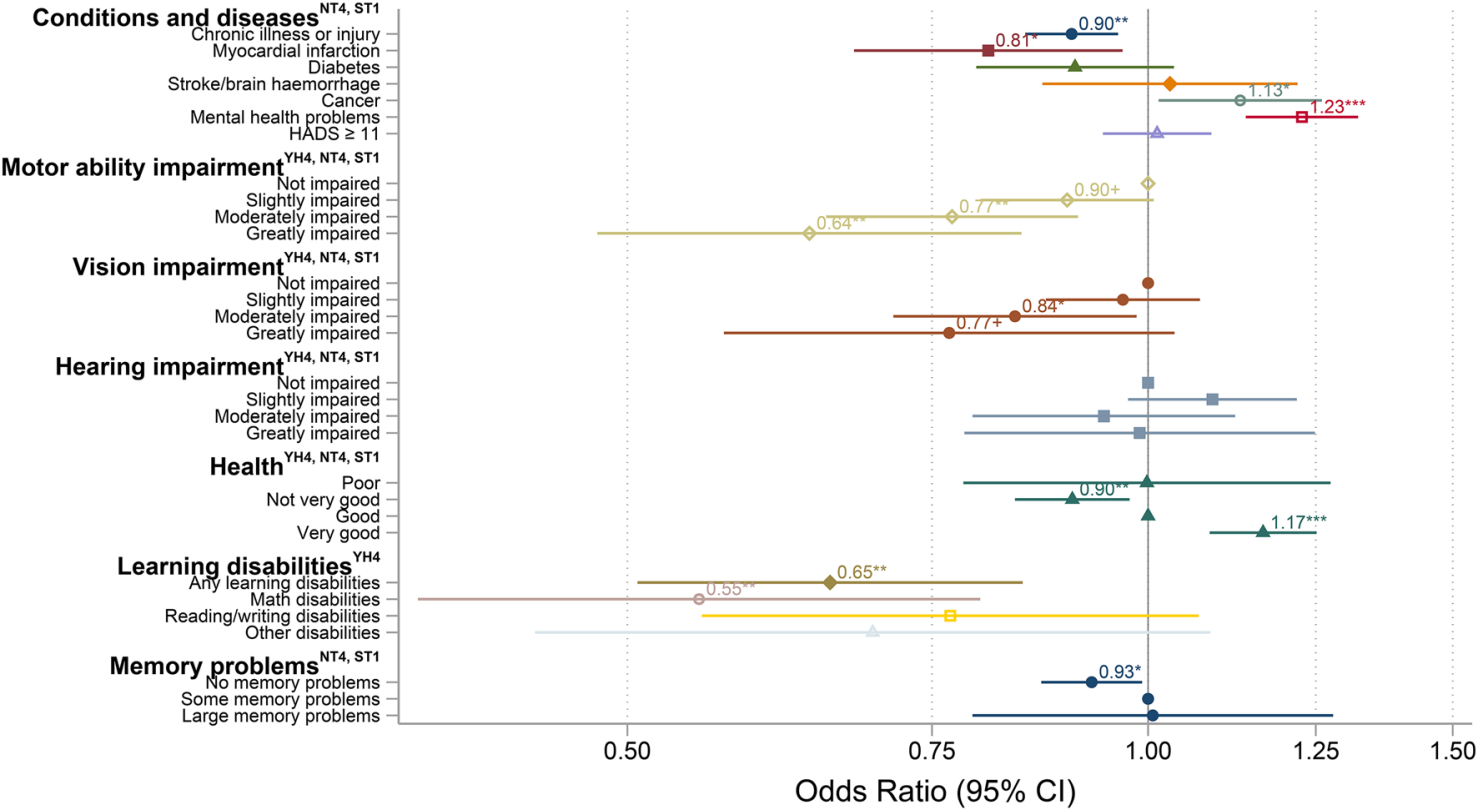
The influence of self-reported somatic and mental health problems, as well as learning disabilities and self-reported memory problems in HUNT4 on the probability of participating in Hjernetrim. Each color represents a separate model. Fifty-year-olds, women, and those with secondary education were used as references as these were the most numerous subgroups. For details on variables and their frequency, see Tables 1 and 2.

The likelihood of participation in Hjernetrim was lower among those reporting a chronic disease or injury (OR = 0.90, 95% CI [0.85, 0.96]), having had a myocardial infarction (OR = 0.81, 95% CI [0.67, 0.97]), motor impairment (moderately impaired: OR = 0.77, 95% CI [0.65, 0.91]; greatly impaired: OR = 0.64, 95% CI [0.48, 0.84]) and a vision impairment (moderately impaired: OR = 0.84, 95% CI [0.71, 0.98]). On the other hand, those who had or had had cancer (OR = 1.13, 95% CI [1.01, 1.26]) and mental health problems (OR = 1.23, 95% CI [1.13, 1.32]) had a higher likelihood of participating. Diabetes, stroke, HADS score of 11 or higher, and hearing impairment had no significant association with participation. Compared to those who reported good health (the most numerous group) at the time of HUNT4, those with not very good health were less likely (OR = 0.90 CI [0.84, 0.98]), and those with very good health were more likely (OR = 1.17, 95% CI [1.09, 1.25]) to participate in Hjernetrim. An overview of the ORs for all the aforementioned variables is presented in Fig. 4.

#### Self-reported cognitive variables associated with partaking in Hjernetrim

The likelihood of participation in Hjernetrim among teenagers was lower in those with learning disabilities (OR = 0.65, 95% CI [0.51, 0.85]), especially math disabilities (OR = 0.55, 95% CI [0.38, 0.80]). In adults, participation was lower in those reporting no memory problems (OR = 0.93, 95% CI [0.87, 0.99]). An overview of OR for participation relative to self-reported cognition is presented in Fig. 4.

#### Lifestyle satisfaction, smoking, and alcohol use

**Figure 5:**
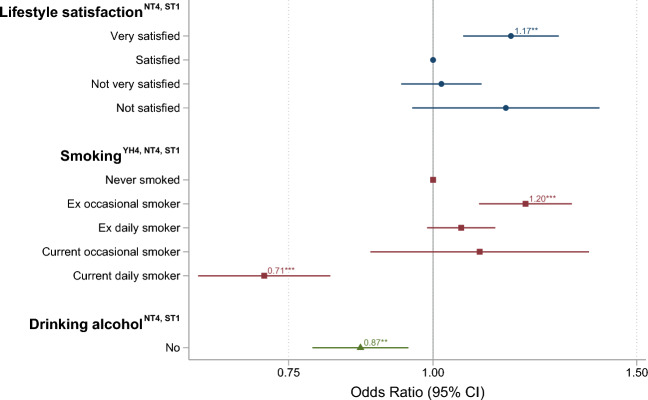
The influence of self-reported lifestyle factors in HUNT4 on the odds ratio of participating in Hjernetrim. Each color represents a separate model. Fifty-year-olds, women, and those with secondary education were used as references as these were the most numerous subgroups. For details on variables and their frequency, see Tables 1 and 2.

Compared to those who were satisfied with their lifestyle (diet, exercise, smoking and drinking habits), those who were very satisfied were more likely to participate (OR = 1.17, 95% CI [1.06, 1.28]) (Fig. 5). Compared to those who never smoked, ex-occasional smokers were more likely (OR = 1.20, 95% CI [1.10, 1.32]), whereas current daily smokers were less likely (OR = 0.71, 95% CI [0.62, 0.81]) to participate. Those who abstain from alcohol were less likely to participate (OR = 0.87, 95% CI [0.79, 0.95]).

#### Demographics of Hjernetrim participants compared to the general population

This analysis compared the distribution of age and sex in those who participated in Hjernetrim and everybody else in the catchment area, i.e., both those participating in the HUNT4 study and not. These results were quite consistent with the above results, showing the 60–69-year group to be the most likely (OR = 1.20, 95% CI [1.11, 1.31]), and the ≥ 80-year-olds to be the least likely (OR = 0.20, 95% CI [0.16, 0.24]) to participate in Hjernetrim. Men were less likely to participate (OR = 0.71, 95% CI [0.67, 0.75]) than women. The distribution of age and sex among Hjernetrim participants and those in the catchment area who did not participate is presented in Supplementary Table S1. The full results of logistic regression are presented in Supplementary Table S2.

### Hjernetrim engagement

#### Computer familiarity, hardware, and software

The majority of Hjernetrim participants rated themselves as familiar with computers (Table 2, Supplementary Table S3), with 90.6% of men and 89.2% of women reporting being rather comfortable or very comfortable using them.

Being younger, male, and having more education were associated with higher computer familiarity (Supplementary Table S4).

**Figure 6:**
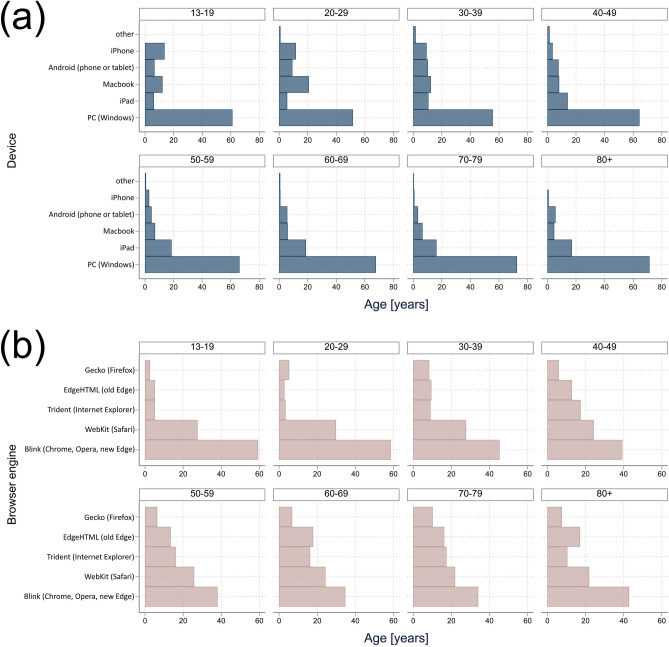
Distribution of devices (**a**) and browser engines (**b**) used to log on to the web-based platform, subdivided by age group.

Personal computers running Windows were the most frequently used devices, accounting for 52.1–73.6% of devices across all age groups. The frequency of the other devices in descending order was: iPads (5.9–18.8% across age groups), Macbooks (5.1–20.7%), Android devices (3.3–9.4%), iPhones (0.7–12.0%), and other devices (0.0–1.7%). The distribution of devices used varied with age (Fig. 6a).

Blink (used in Google Chrome, Opera, and recent versions of Microsoft Edge) was the most frequent browser engine across all the age groups (Fig. 6b). Blink dominated the most in the 13–19 age group (59.4%) but was less frequent in the 70–79-year group (34.1%). The frequency of the other browser engines in descending order was: WebKit (Safari, 21.9–29.9% across age groups), Trident (Internet Explorer, 3.4–17.4%), EdgeHTML (older versions of Microsoft Edge, 2.8–17.9%), and Gecko (Mozilla Firefox, 2.6–10.2%). The relative popularity of browser engines other than Blink and WebKit increased with age (Fig. 6b).

#### Day and time of participation

Thursdays were the most popular days to participate in most age groups, with 17.6% of users starting the battery on that day, followed by Tuesdays (16.8%), Mondays (16.1%), Wednesdays (15.1%), Fridays (13.2%), Sundays (12.2%), and Saturdays (9.1%).

**Figure 7:**
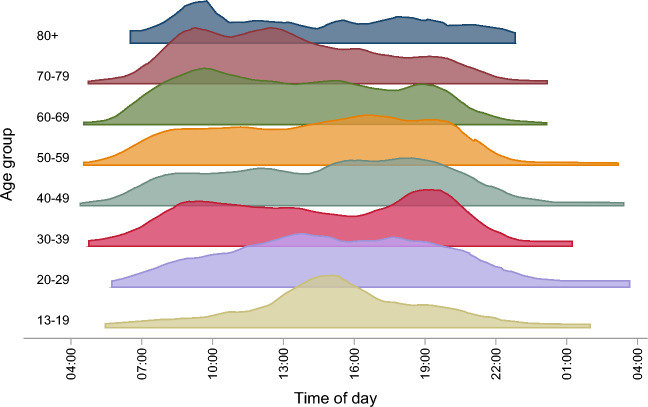
The distribution of Hjernetrim participants’ logging into the battery during the day by age group. The Y axis illustrates the participant density of each age group.

The mean time of starting the battery was 14:10, with 90% of participants starting between 7:33 and 20:42. Preferred starting times varied widely across different age groups. The data showed that younger participants generally started in the afternoon, those in working age tended to start in the evenings, while older adults preferred starting in the mornings (Fig. 7).

#### Time spent

**Figure 8:**
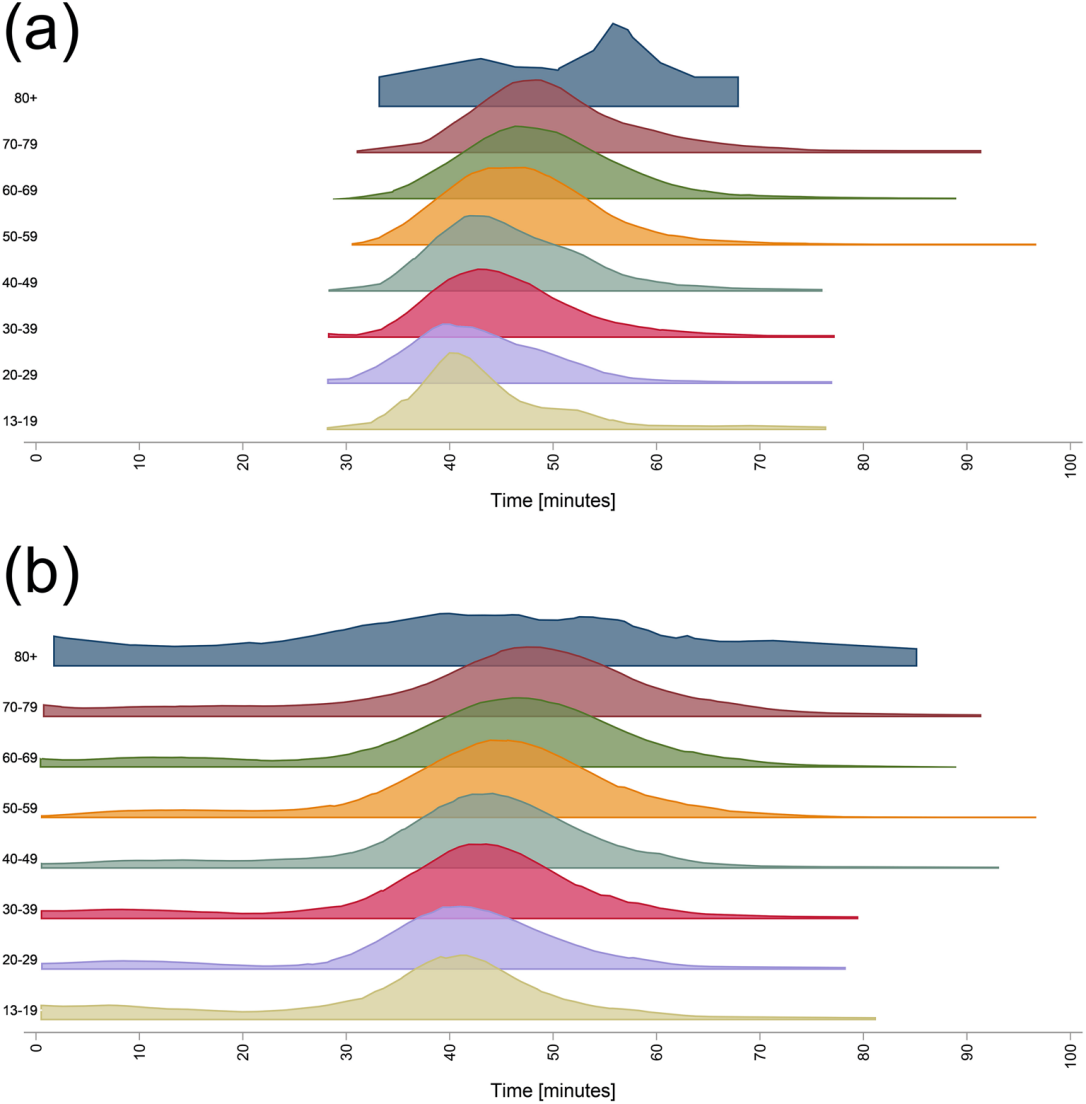
Time spent in Hjernetrim in (**a**) those who completed all tasks and (**b**) regardless of battery completion. The Y axis illustrates the participant density of each age group.

For participants who completed all the tasks, the mean duration was 47 min, with a median of 46 min and a range from 27.6 to 96.7 min (Fig. 8a). The time to complete the whole battery increased linearly with age (t = 18.35, p < 0.001), with a mean increase of time of about 1 min per 7 years of age. When including participants who discontinued, the average time spent was 42 min, with a range from 0.2 to 96.7 min and a median of 44 min (Fig. 8b).

#### Completion and discontinuation of test battery

**Figure 9:**
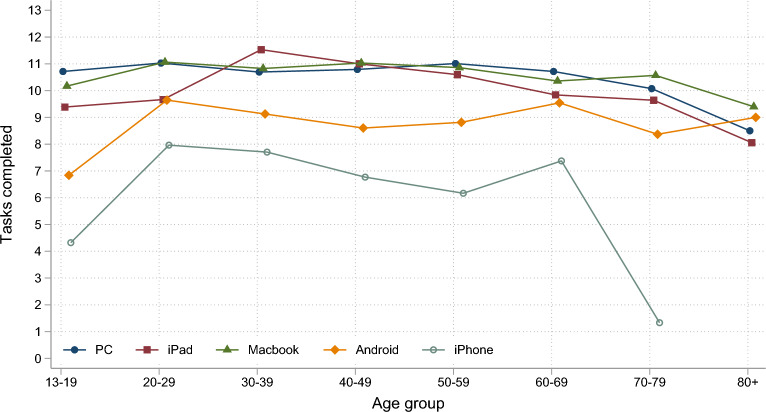
The mean number of completed tasks in Hjernetrim by device and age group. Frequencies below 5 and observations of unidentified hardware were not included in the figure.

The completion rate was high with 58% (n = 3248) completing all tasks and an additional 22.5% (n = 1270) completed 7 tasks or more. A total of 13.2% (n = 743) participants completed < 7 tasks, while 6.7% (n = 373) did not complete any tasks. The mean number of completed tasks was 10.3 out of 13 tasks. Several factors influenced task completion (*R*^2^ = 0.06, *F*(16, 5313) = 21.28, *p* < 0.001), including education, age, and device (Table 3). Participants with tertiary education were more likely to complete all tasks compared to those with secondary education. The number of completed tasks was lower in older adults. Sex and computer familiarity were not related to the number of tasks completed, but using an Android device, an iPhone or an iPad negatively influenced completion rates (Fig. 9).

**Table 3:** Results of linear regression examining the association between sex, education, age group, device and computer familiarity on the number of completed tests.

	Coefficient	SE	t	*p*-value	95% CI	
Sex						
Female	(base)					
Male	− 0.10	0.12	− 0.80	0.402	− 0.34	0.14
Education						
Primary	0.08	0.26	0.30	0.744	− 0.42	0.59
Secondary	(base)					
Tertiary	0.73	0.13	5.60	** < 0.001**	0.47	0.98
Age group						
13–19	− 0.47	0.27	− 1.70	0.085	− 1.00	0.07
20–29	0.16	0.24	0.70	0.502	− 0.31	0.63
30–39	− 0.01	0.22	0.00	0.976	− 0.44	0.43
40–49	− 0.22	0.20	− 1.10	0.279	− 0.62	0.18
50–59	(base)					
60–69	− 0.33	0.18	− 1.80	0.074	− 0.68	0.03
70–79	− 0.87	0.20	− 4.30	** < 0.001**	− 1.27	− 0.47
80 +	− 2.17	0.46	− 4.70	** < 0.001**	− 3.09	− 1.26
**Device**						
PC (windows/Mac)	(base)					
iPad	− 0.46	0.17	− 2.70	**0.007**	− 0.80	− 0.13
MacBook	− 0.19	0.21	− 0.90	0.349	− 0.60	0.21
Android (smartphone/tablet)	− 1.78	0.25	− 7.20	** < 0.001**	− 2.27	− 1.29
iPhone	− 4.22	0.30	− 14.20	** < 0.001**	− 4.81	− 3.64
Other	− 0.01	0.61	0.00	0.982	− 1.21	1.19
Computer familiarity	0.08	0.08	1.00	0.316	− 0.07	0.23

Likewise, those with tertiary education were less likely to discontinue the battery (OR = 0.72, 95% CI [0.59, 0.88]), while participants who used Android devices (OR = 1.63, 95% CI [1.10, 2.11]) and iPhones were more likely to discontinue (OR = 2.17, 95% CI [1.49, 3.17]). Sex and computer familiarity were not related to discontinuation.

The test associated with this greatest discontinuation was the learning trials of the Verbal Memory task (n = 249, corresponding to 41% of all such cases and 4% of all participants).

#### Participation at field stations

Overall, 23 (0.4%) participants performed the battery in field stations. Their mean age was 73 years (49.9–85.6 years), which is significantly higher than the rest of the sample (t = 6.06, *p* < 0.001). Those participants completed fewer tasks on average compared to the entire sample (8.6 vs 10.3), but the difference was not statistically significant (t = − 1.07, *p* = 0.284), likely due to the low number of field station participants and large variability of the number of tasks they completed. Participants at the field stations spent an average of 52.7 min (19.5–89.9 min) on Memoro tasks, over 10 min longer than those who solved them at home (t = 3.10, *p* = 0.002).

#### User experience and feedback for participants

A total of 27.6% reported being interrupted or disturbed on at least one task. A total of 58.1% answered the open question about their experience with the test battery. Participants were more likely to leave a comment if they finished all the tasks compared to those who finished most (OR = 0.65, 95% CI [0.56, 0.76]), less than half (OR = 0.006, 95% CI [0.003, 0.010]), or none of the tasks (OR < 0.001, 95% CI [< 0.001, 0.001]), and the probability increased with age between 20 and 80 years. Conversely, the group aged 13–19 was significantly more likely, while the 80 + age group was significantly less likely to provide comments on their experience compared to their neighboring age groups. Sex and education did not influence the likelihood of leaving comments. An overview of the comments stratified by topics is presented in Table 4.

**Table 4:** Overview of the user experiences reported by participants at the end of testing stratified into five different topic groups.

User experience	Sex		Total
	Female	Male	
No comments^1^	1331 (40.9%)	1029 (43.2%)	2360 (41.9%)
General positive experience	1238 (38.1%)	891 (37.4%)	2129 (37.8%)
Comments about difficulty	249 (7.7%)	200 (8.4%)	449 (8.0%)
General negative experience	177 (5.4%)	111 (4.7%)	288 (5.1%)
Any technical issue	110 (3.4%)	48 (2.0%)	158 (2.8%)
Other comments	149 (4.5%)	101 (4.2%)	250 (4.4%)

Results are provided as total numbers and percentages. See materials and methods for more on topic of stratification.

^1^Out of those who left no comments, 1526 (64.7%) did not finish all the tasks. See materials and methods for more on topic stratification.

Only 20.4% (n = 1147) logged back into the Memoro website to access their performance results and an additional 0.8% (n = 44) contacted us directly to receive their results.

## Discussion

The study had two main objectives. The first objective was to compare the characteristics of individuals who participated in the HUNT4-Hjernetrim cognitive substudy with those who did not, focusing on sociodemographics, self-reported health, self-reported cognition, and lifestyle. The second objective was to evaluate participants' engagement with the study with regards to their computer familiarity and preferred device and browser; the time of day and week they took the test; the time spent completing the tasks; the number of tasks completed and reasons for discontinuing; participation at field stations; and their self-reported user experience and the frequency with which they accessed their performance results.

To our knowledge, this is the first study to examine participation bias and engagement in web-based cognitive testing in a general population. Our study provides insight into sociodemographic, health, self-reported cognition, and lifestyle characteristics of those opting in and completing web-based cognitive testing. The overall participation rate was only 8.6% of those invited and 4.7% of the general population, suggesting substantial room for improvement in how participants are invited, re-invited and reminded of such studies. Importantly, the field stations were an ineffective means of recruitment. Those self-selecting into web-based testing in HUNT4 were predominantly between 50 and 80 years old and had tertiary education. Web-based testing appeared to appeal more to participants living alone and not working, and those with self-reported good health, certain diagnoses (e.g., cancer, mental health) and subjective experienced cognitive issues among adults, but not to teenagers with learning problems. To reach all age groups, web-based, unsupervised cognitive testing needs to be available on a multi-device platform. Finally, when participants did log in and engage, most completed all tasks and reported a positive experience. The number of completed tasks was lower and the risk of discontinuation was higher among older participants, those without tertiary education, and those using phones or tablets rather than PCs. Weekdays were optimal for participation.

### Characteristics of Hjernetrim participants

#### Sociodemographics

Hjernetrim participants were between 13 and 97 years of age, illustrating that web-based testing can be used to recruit participants of all age groups. The age distribution in Hjernetrim participants was comparable to those who only participated in HUNT4, but with the highest participation rate among those aged 50–69 years, whereas participants above 80 years were relatively underrepresented in Hjernetrim.

Hjernetrim participation rates were higher with age until the late sixties in women and seventies in men and lower thereafter in both sexes compared to only HUNT4 participation. This result differs from previous health-related studies, for example in heart failure RCTs and a study of self-selection for digital health research participants tended to be younger than non-participants^15–17^. The higher participation rate among middle-aged and older (but not oldest old) in Hjernetrim could be attributed to more leisure time at these ages^18^. Another explanation might be that people become more invested in their cognitive health as they age, which makes them more likely to view involvement in this type of research as advantageous for themselves or their age group at large^19^. Our finding of a lower participation rate among those who reported having no memory problems supports this notion.

Nevertheless, the least likely to participate in Hjernetrim were those aged 80 years and above. This might be due to the general decrease in engagement in leisure activities despite more free time, observed in the oldest old^20^, which could have affected Hjernetrim more than the main study because of the latter’s somatic health testing with feedback and visit to a test clinic with its social aspect. The relatively low participation rate among the older participants could also be due to another substudy, HUNT4 70 + in which MoCA and other cognitive tests were performed^21^. The number of 80- and 90-year-olds in Hjernetrim compared to HUNT4 could also be due to the recruitment procedure. Many of the older participants in HUNT4 have taken part in previous HUNT waves and are familiar with this part of the study while web testing is something new to them. The computer skills assumed to be required to participate could have been another barrier. The high computer familiarity observed in Hjernetrim participants (see Hjernetrim engagement) supports this notion. Hjernetrim was initially developed and validated for older adults, and they were able to follow instructions irrespective of computer familiarity level^3^. However, we cannot rule out that the need to enter the website address and login credentials manually, which was required here, could have discouraged individuals from participating. Using passwordless authentication, for example by sending an electronic invite via text or e-mail to participants that contains a participant-specific Magic Link allowing direct access to the battery without manually entering login credentials, would likely increase participation rates across all ages^22^.

Men were less likely to participate than women. This is a well-known bias in epidemiological studies^23–25^ and even more pronounced than in this study in other self-administered and self-recruiting cognitive test batteries such as MindCrowd^26^, BAM-COG^27^, and CFT^28^. The reason behind this sex difference is unclear, but it has been attributed to women being more engaged in volunteering, as shown in the previous HUNT waves^5,13^. Intriguingly, the trend was reversed in older adults partaking in Hjernetrim, with more men than women participating after the age of 65. A potential explanation is lower computer usage and computer self-efficacy in women over the age of 65, reported by Statistics Norway (ssb.no/en/statbank/table/11124) which was also reflected in the Hjernetrim participants (Supplementary Table S2). If the same was true for the entire cohort, it could be assumed that less familiarity with computers discouraged older women from participating in the study. As younger generations show high computer self-efficacy and no sex differences^29^, we predict increasingly higher rates of participation in web-based studies among elders, including women, in the future. It is interesting that although women across all age groups indicated lower familiarity with computers compared to men, still a greater number of women under the age of 65 participated in the Hjernetrim study. This implies that factors not accounted for in the study may have contributed to the observed differences in participation between men and women.

The Hjernetrim participants were highly educated, with approximately 54% having tertiary education. This percentage was higher than in the HUNT4 study and consistent across age and sex. High educational attainment is a common selection bias across study designs^11,17,24,25,30,31^, and also reported in other large web-based cognitive studies such as MindCrowd where the majority had between 14 and 16 years of education^32^. Highly educated people tend to exhibit different engagement patterns (as discussed below) and achieve higher cognitive scores^33^ compared to less educated participants. This overrepresentation might limit the generalizability and could skew the average test performance, task engagement, and user experience, impacting the study's overall results. This bias could have been minimized by sending reminders to those receiving letters and not participating, which we were not allowed as per the HUNT Research Centre regulations.

Participants living alone were more likely to participate in Hjernetrim. Reaching those people is especially important in cognitive ability studies since it was demonstrated that those who are lifelong single or widowed have a higher risk of dementia^34^ and that singlehood was associated with negative outcomes such as somatic symptoms, anxiety and insomnia, severe depression, and romantic loneliness^35^.

Living in urban areas significantly increased the probability of participation and the effect was significant across age and education groups. It is not clear why living in rural areas decreased the probability of participation given that the opposite effect was observed for participation in the HUNT4 study^13^.

Not being occupationally active increased participation in the study, particularly among individuals between 50 and 70 years of age. This trend is likely due to the more leisure time, which could facilitate greater participation. These findings indicate that middle-aged individuals who are not engaged in occupational activities represent a key demographic for web-based cognitive studies. Additionally, this group is known to be at a heightened risk for various somatic and mental health issues^36–38^. Our study suggests that online research methodologies could be an effective approach to engage and study this demographic, potentially addressing their unique health challenges.

#### Somatic and mental health

The odds ratios for participation were impacted by different health conditions in varying ways. The lack of influence of diabetes, stroke, hearing impairment, and HADS score over 11 on participation is probably important to note as it shows that somatic and mental health challenges do not necessarily impair the ability to take part in web-based cognitive testing. This is further exemplified by the increased participation odds in those reporting cancer, or mental health problems they sought help for. Those conditions have been connected to cognitive problems or complaints^39–42^. One could speculate that facing such challenges would make these groups more conscious about their cognitive health and thus more likely to participate, suggesting that web-based testing is a suitable method for studies targeting those groups, although previous studies showed otherwise^17^. The higher participation rates among those with mental health problems they sought help for, provide support for this reasoning. On the other hand, we observed lower participation among groups of individuals who reported “not very good” health or experienced impairment due to chronic illness or injury, myocardial infarction, or motor and vision impairments. Depending on the type of injury or chronic disease, participants could need physical therapy or treatments, and thus not have enough time or possibility to participate in this study. Likewise, participants with motor or vision impairment could have difficulties using the devices or perform the tasks. Implementation of universal design will be important to meet the needs of those with sensory and motor deficits in the future. A shorter test battery might have been better as it would reduce the overall load on participants and facilitate their participation.

Overall, in a study targeting the general population, no clear pattern emerged suggesting that diseases or impairments necessarily lead to reduced participation. Instead, the data suggest a more complex picture, with some diseases, disorders or impairments associated with higher participation rates and others associated with lower participation rates, similar to a previous study examining HUNT participation^43^. Additionally, some groups previously considered hard to reach, such as those living alone^44^ and those not occupationally active appear to have a high participation rate in a study with a web-based design.

#### Self-reported cognition

Teenagers with learning disabilities, especially in mathematics, participated less often. Since learning difficulties negatively impact academic well-being^45^, students with learning disabilities could have felt discouraged from participating in cognitive testing. On the other hand, adults who self-reported having no memory problems were less likely to participate, which again could indicate that worrying about one’s health increases participation odds. It should be noted that subjective memory complaints in this cohort were connected with lower scores on the verbal list learning test^46^, indicating correspondence between subjective and objective measures of cognition. The dichotomy in participation between teenagers and adults with self-reported cognitive issues indicates that different strategies might be needed when recruiting different age groups to cognitive testing.

#### Lifestyle satisfaction, smoking, and alcohol use

Being very satisfied with one´s lifestyle (diet, exercise, smoking and drinking habits) increased participation. This is a largely expected outcome given that those who tend to lead a healthier lifestyle and are satisfied with life show other characteristics associated with higher participation in our study, such as higher education, urban place of residence, and less or no chronic diseases^47^.

Individuals who formerly smoked occasionally were more likely to participate, whereas current daily smokers and those who abstain from alcohol were less likely to take part. These findings resemble those of the UK Biobank^24^, in that in both studies, participants were less likely to be current smokers and never drinkers compared to nonrespondents from the general population. This pattern has also been seen in a phone-based Stockholm Health of the Population Study^48^.

Taken together, those who self-selected to Hjernetrim constitute only a small part of the general population and are not fully representative of the general population with regard to several sociodemographic attributes, somatic and mental health, cognition, and lifestyle factors. The relatively low participation rate was partly caused by the fact that only those who participated in the main HUNT4 study were invited and by regular mail. Still, the rate was comparable to other general population studies such as the UK Biobank^24^. Among the largest and expected sources of bias were age, educational attainment, self-reported health and certain health conditions such as impaired motor ability. However, contrary to the popular “healthy user bias”^7,49–51^, certain conditions such as cancer and self-reported mental health problems actually increased participation. Moreover, most of the assessed factors, including life-altering ones such as vision and hearing impairment, had relatively small to no effect on participation. Lastly, it is important to note that while self-selection bias is a widespread concern in health research, it does not necessarily compromise the generalizability of the results, as long as its sources are clearly understood and factored into the interpretation of the study's findings^7,44,52^. It can also be accounted for by using certain statistical approaches, for example by using inverse probability weighting^53^.

### Hjernetrim engagement

#### Computer familiarity, hardware, and software

In computerized studies such as Hjernetrim, computer familiarity influences participation^54,55^. Our results confirm that the vast majority of those who self-select to web-based studies were comfortable or very comfortable with using computers. This was expected since 95% of people in Norway use the internet daily or almost every day, including more than 75% of those ages 65–79 (ssb.no/en/statbank/table/11124). However, computer familiarity was lower in women and decreased with age, which could have affected the results^56^. Furthermore, since computer familiarity was high in people younger than the late seventies, this issue was limited. An increase in computer familiarity is expected in the future, and a Dutch study reported that less than 5% of older participants did not use a computer frequently^57^. A Malaysian study found that 77% of the sample of older adults had computer-related expertise and only 3% of the sample had none^58^. Similarly, internet usage in Norway has consistently increased among people of all age groups over the last decades. For example, the percentage of individuals aged 55–74 who reported using the internet in the past 3 months surged from 47.8% in 2005 to 99.1% in 2023 (stats.oecd.org/Index.aspx?DataSetCode = ICT_HH2). Overall, computer familiarity seems not to be an issue for most participants and will likely become even less of a concern in the future.

As recommended, most (74%) of the participants used PCs (Windows PC or Macbook) to partake in Hjernetrim. Among those who did not follow this advice, younger participants predominantly chose phones, whereas older participants were more inclined to use tablets. This highlights the need for a web-based platform to be accessible and optimized for all devices to prevent the loss of potential participants. We cannot rule out that we lost participants who did not or could not participate on a computer. Importantly, performing the tests on a phone was associated with a lower rate of completed tasks and a higher likelihood of discontinuation compared to a Windows PC. Participants will experience more disruptions using their mobile devices as the web-based format does not hinder pop-ups or calls. Additionally, conducting tests on smaller screens might present more challenges. Considering that mobile devices are the most commonly used platform for internet access in general (perficient.com/insights/research-hub/mobile-vs-desktop-usage; gs.statcounter.com/platform-market-share/desktop-mobile-tablet) and become the preferred computing device across various settings^59,60^, it is crucial to implement methods for obtaining cognitive data of the same quality irrespective of the type of device.

The Blink browser engine, integral to the recommended Google Chrome browser, emerged as the most popular choice among all age groups, again implying that participants generally adhered to the guidelines. The selection of a browser is primarily a concern for the participant’s security^61,62^; thus, educating participants about this aspect could enhance compliance. However, there may be reluctance among some participants, particularly older adults, to download or use an unfamiliar browser. Our results support this as the use of older and non-standard browsers increased with age. A hesitancy to update the browser could potentially impact participation rates.

#### Day and time of participation

The preferred time of participation appeared to depend on the convenience of a certain time, i.e., finding a day and time when it was possible to devote ≥ 40 min to taking part in the online testing, and when the different age groups feel their mental capacity peaks at different times during the day, morning for older adults, later in the day for teenagers, and evenings for working age participants. Importantly, while participation at a preferred time does not affect the results in all age groups, it can help older adults perform to the best of their ability^63,64^. Web-based methodology is optimal in this respect. Interestingly, there was a significant variation in participation rates throughout the week. Thursdays and Tuesdays emerged as the most preferred days for participation, while Saturdays and Sundays saw the lowest engagement across almost all age groups. This finding is crucial for planning future web-based study promotions, indicating that advertising efforts might be more effective if concentrated on the weekdays. This conclusion is inconsistent with some previous studies on the impact of invitation day on response rate to online questionnaires^65,66^, but aligns with insights from studies conducted by marketing and survey platforms, as evidenced by the data and recommendations shared on their respective blogs (such as checkmarket.com/blog/survey-invitations-best-time-send, getresponse.com/blog/best-time-to-send-email-infographic, zendesk.com/blog/maximize-survey-response-rates, mailchimp.com/resources/insights-from-mailchimps-send-time-optimization-system).

#### Time spent

The average time participants spent completing the tests was approximately 46 min, exceeding the 40-min duration outlined in the informational leaflet. This extended duration can be partially attributed to the high performance of participants^46,67^. For example, successfully recalling more words in a verbal memory test results in a longer completion time, while failing a training task could result in skipping the main part of that task. The fact that most participants completed the majority or all of the tasks and provided predominantly positive feedback suggests that a duration of over 40 min is generally acceptable. Yet, it is notable that some individuals, particularly older adults, spent considerably more time than expected, which can lead to participant fatigue, even if it does not decrease performance^67,68^. Therefore, future studies should aim to strike a balance between the length of the tests and the number of cognitive domains assessed, possibly across multiple sessions, to optimize both participation rates and completion rates without inducing fatigue.

#### Completion and discontinuation of test battery

Overall, the completion rate for all tests was high for Hjernetrim participants. It is important to note that participants who completed fewer tests or discontinued the battery shared some demographic characteristics with those who were less likely to participate in the study overall, such as lacking tertiary education and, in the case of those who completed fewer tests, being in the oldest age groups. This suggests that successfully recruiting harder-to-reach individuals for web-based studies does not guarantee complete data. A similar pattern was observed in a previous web-based depression intervention, where lower education levels increased the risk of participants dropping out before completing most of the study modules. This risk was further heightened by younger age and being male^69^. However, we did not observe this in our study, possibly due to differences in the study topics. Additionally, longer or more demanding tasks, such as verbal list learning, appeared to cause relatively high dropout rates. This finding is consistent with previous research that demonstrated an increase in participant discontinuation with longer survey lengths in several web-based psychological questionnaire studies involving undergraduate students^70^. Other verbal memory tests such as verbal paired associates might be a better choice.

#### Participation at field stations

Field stations turned out not to be very popular. Less than 0.5% of all participants participated at a Hjernetrim field station despite advertisements in newspapers, shopping malls, and other HUNT field stations. The participants who chose field stations were predominantly older and not very comfortable with computers, which was most likely the reason they signed up. In theory, field stations could help to recruit important, underrepresented groups, but we were not able to harness such potential. Other solutions, like having the field stations available at the same time as the main HUNT4 data collection was performed, collaborations with senior centres, or help over a remote connection, might have been more efficient.

#### User experience and feedback for participants

Approximately 28% of participants reported disruptions during one or more tasks. Despite this, the average rate of disruption per task was approximately 5%, which we consider relatively low. This suggests that testing participants in non-controlled environments likely had a limited impact on results, a conclusion supported by previous studies suggesting that web-based methodology does not negatively impact the quality of data and study validity or reliability^3,71–73^.

A positive experience was reported by approximately 65% of participants. Negative experience or technical issues were reported by less than 3% of participants, despite the use of a wide range of devices and software. This suggests that most participants found web-based cognitive testing quite enjoyable, in line with previous Hjernetrim study findings^3^. A caveat here is that participants who provided feedback were mainly those completing all tasks.

Finally, most participants did not log back in for feedback on their performance. A potentially more rewarding and effective approach for feedback could be to provide immediate results or send the results via regular mail like the other results in HUNT4.

## Conclusion

This study is the first to examine the characteristics of individuals who volunteered to participate in web-based cognitive testing versus those invited who did not participate and to identify sources of participation bias while also showing in detail participants’ engagement in the study. Our study found that groups underrepresented in prior research, such as individuals with mental health concerns, subjective memory complaints, the unemployed and those living alone, were adequately represented in our sample. Differences in factors like educational attainment, age, and various health conditions contributed to varying selection bias. By recognizing and addressing these sources of bias, we can ensure the representativeness and reliability of our future research results. Moreover, the level of participant engagement observed in our study indicates that web-based cognitive testing is effective, even when the test battery is extensive and time-consuming. Finally, the majority of participants reported having a positive experience with the testing process. The patterns of participant engagement can help refine planning, design, and recruitment strategies for future studies that utilize web-based technologies, ultimately boosting participation rates, data quality and participation experience.

### Supplementary Information

Supplementary Tables.

## Data Availability

The data that support the findings of this study regarding factors affecting the likelihood of participation in Hjernetrim compared to HUNT4 are available on request from the HUNT Databank. HUNT Databank website (https://hunt-db.medisin.ntnu.no/hunt-db) can be used to apply for a research project or order variables. Data on engagement patterns are available from the authors. Access to the data will require review by the regional ethics committee and permission from the HUNT Databank to ensure adherence to GDPR as well as institutional regulations.
