# Supplementary material for: Participation and engagement in online cognitive testing
Source: Sci Rep. 2024 Jun 26;14:14800. doi: 10.1038/s41598-024-65617-w (PMC11208174; doi:10.1038/s41598-024-65617-w)
Supplement: Supplementary file 1 — Supplementary Tables. [file 41598_2024_65617_MOESM1_ESM.docx]

# Supplementary tables

**Supplementary Table 1.** The distribution of age and sex among Hjernetrim participants and those in the HUNT4 study catchment area who did not participate in the HUNT4 study.

| **Age group** | **Non-participants**  **n= 109856** | |  | **Hjernetrim**  **n= 5634** | |
| --- | --- | --- | --- | --- | --- |
|  | **Women** | **Men** |  | **Women** | **Men** |
| 13-19 | 4846 (8.9%) | 5200 (9.4%) |  | 312 (9.6%) | 156 (6.6%) |
| 20-29 | 7787 (14.3%) | 8651 (15.7%) |  | 352 (10.8%) | 159 (6.7%) |
| 30-39 | 6989 (12.8%) | 7336 (13.3%) |  | 389 (12.0%) | 215 (9.0%) |
| 40-49 | 8095 (14.8%) | 8350 (15.1%) |  | 508 (15.6%) | 290 (12.2%) |
| 50-59 | 8183 (15.0%) | 8737 (15.8%) |  | 674 (20.7%) | 437 (18.4%) |
| 60-69 | 7591 (13.9%) | 7922 (14.3%) |  | 631 (19.4%) | 594 (25.0%) |
| 70-79 | 6637 (12.2%) | 6159 (11.2%) |  | 353 (10.9%) | 465 (19.5%) |
| 80+ | 4468 (8.2%) | 2905 (5.3%) |  | 35 (1.1%) | 64 (2.7%) |
| Total | 54596 (100.0%) | 55260 (100.0%) |  | 3254 (100.0%) | 2380 (100.0%) |

**Supplementary Table 2.** Results of logistic regression examining the effects of sex and age group on the likelihood of participation in Hjernetrim compared to those living in the HUNT4 catchment area, invited but declining to participate in the HUNT4 study.

|  | **Odds ratio** | **SE** | **z** | ***p-value*** | **95% CI** | |
| --- | --- | --- | --- | --- | --- | --- |
| **Sex** |  | | | | | |
| Female | (base) | | | | | |
| Male | 0.71 | 0.02 | -12.43 | **<0.001** | 0.67 | 0.75 |
| **Age group** |  | | | | | |
| 13-19 | 0.71 | 0.04 | -6.07 | **<0.001** | 0.63 | 0.79 |
| 20-29 | 0.47 | 0.03 | -13.64 | **<0.001** | 0.43 | 0.53 |
| 30-39 | 0.64 | 0.03 | -8.58 | **<0.001** | 0.58 | 0.71 |
| 40-49 | 0.74 | 0.04 | -6.4 | **<0.001** | 0.67 | 0.81 |
| 50-59 | (base) | | | | | |
| 60-69 | 1.20 | 0.05 | 4.31 | **<0.001** | 1.11 | 1.31 |
| 70-79 | 0.97 | 0.05 | -0.72 | 0.469 | 0.88 | 1.06 |
| 80+ | 0.20 | 0.02 | -15.36 | **<0.001** | 0.16 | 0.24 |

Base refers to reference group, i.e. the most numerous group.

**Supplementary Table 3.** The distribution of computer familiarity across sex and age groups.

| **Age group** | **Computer familiarity [mean (SD)]** | | |
| --- | --- | --- | --- |
|  | **Women** | **Men** | **Total** |
| 13-19 | 4.56 (0.62) | 4.62 (0.77) | 4.58 (0.68) |
| 20-29 | 4.76 (0.56) | 4.81 (0.44) | 4.78 (0.53) |
| 30-39 | 4.69 (0.59) | 4.68 (0.67) | 4.68 (0.62) |
| 40-49 | 4.53 (0.71) | 4.61 (0.67) | 4.56 (0.70) |
| 50-59 | 4.37 (0.84) | 4.40 (0.78) | 4.38 (0.82) |
| 60-69 | 4.15 (0.84) | 4.25 (0.83) | 4.20 (0.84) |
| 70-79 | 3.96 (0.88) | 4.14 (0.82) | 4.06 (0.85) |
| 80+ | 3.41 (1.35) | 3.70 (1.05) | 3.60 (1.17) |
| Total | 4.40 (0.8) | 4.39 (0.80) | 4.39 (0.80) |

Computer familiarty was scored on a 5-point scale,

with 1. Very uncomfortable; 2. Rather uncomfortable;

3. Neither; 4. Rather comfortable; 5. Very comfortable

**Supplementary Table 4**. Results of ordered logistic regression examining how sex, age group and education were associated with computer familiarity among Hjernetrim participants.

|  | **Coefficient** | **SE** | **z** | ***p-value*** | **95% CI** | |
| --- | --- | --- | --- | --- | --- | --- |
| **Sex** |  | | | | | |
| Female | (base) | | | | | |
| Male | 0.26 | 0.06 | 4.55 | **<0.001** | 0.15 | 0.38 |
| **Age group** |  | | | | | |
| 13-19 | 1.20 | 0.13 | 9.16 | **<0.001** | 0.94 | 1.46 |
| 20-29 | 1.50 | 0.13 | 11.22 | **<0.001** | 1.24 | 1.76 |
| 30-39 | 0.90 | 0.12 | 7.78 | **<0.001** | 0.67 | 1.13 |
| 40-49 | 0.40 | 0.10 | 4.06 | **<0.001** | 0.21 | 0.59 |
| 50-59 | (base) | | | | | |
| 60-69 | -0.54 | 0.08 | -6.50 | **<0.001** | -0.71 | -0.38 |
| 70-79 | -0.90 | 0.09 | -9.80 | **<0.001** | -1.08 | -0.72 |
| 80+ | -1.68 | 0.21 | -7.92 | **<0.001** | -2.09 | -1.26 |
| **Education** |  |  |  |  |  |  |
| Primary | -0.43 | 0.12 | -3.60 | **<0.001** | -0.66 | -0.20 |
| Secondary | (base) | | | | | |
| Tertiary | 0.86 | 0.06 | 14.02 | **<0.001** | 0.74 | 0.97 |

Base refers to reference group, i.e. the most numerous group.
